# Supplementary material for: Single-cell RNA-seq of Drosophila miranda testis reveals the evolution and trajectory of germline sex chromosome regulation
Source: PLoS Biol. 2024 Apr 30;22(4):e3002605. doi: 10.1371/journal.pbio.3002605 (PMC11135767; doi:10.1371/journal.pbio.3002605)
Supplement: S1 Fig — (A) UMAP projection of all UMI clusters. (B) Expression of maker genes used to identify cell types across clusters. The group of genes expressed in the spermatocyte necessary for meiosis (collectively known as the meiotic arrest genes) are labeled in red. The data underlying this figure can be found in S1 Data. (PDF) [file pbio.3002605.s004.pdf]

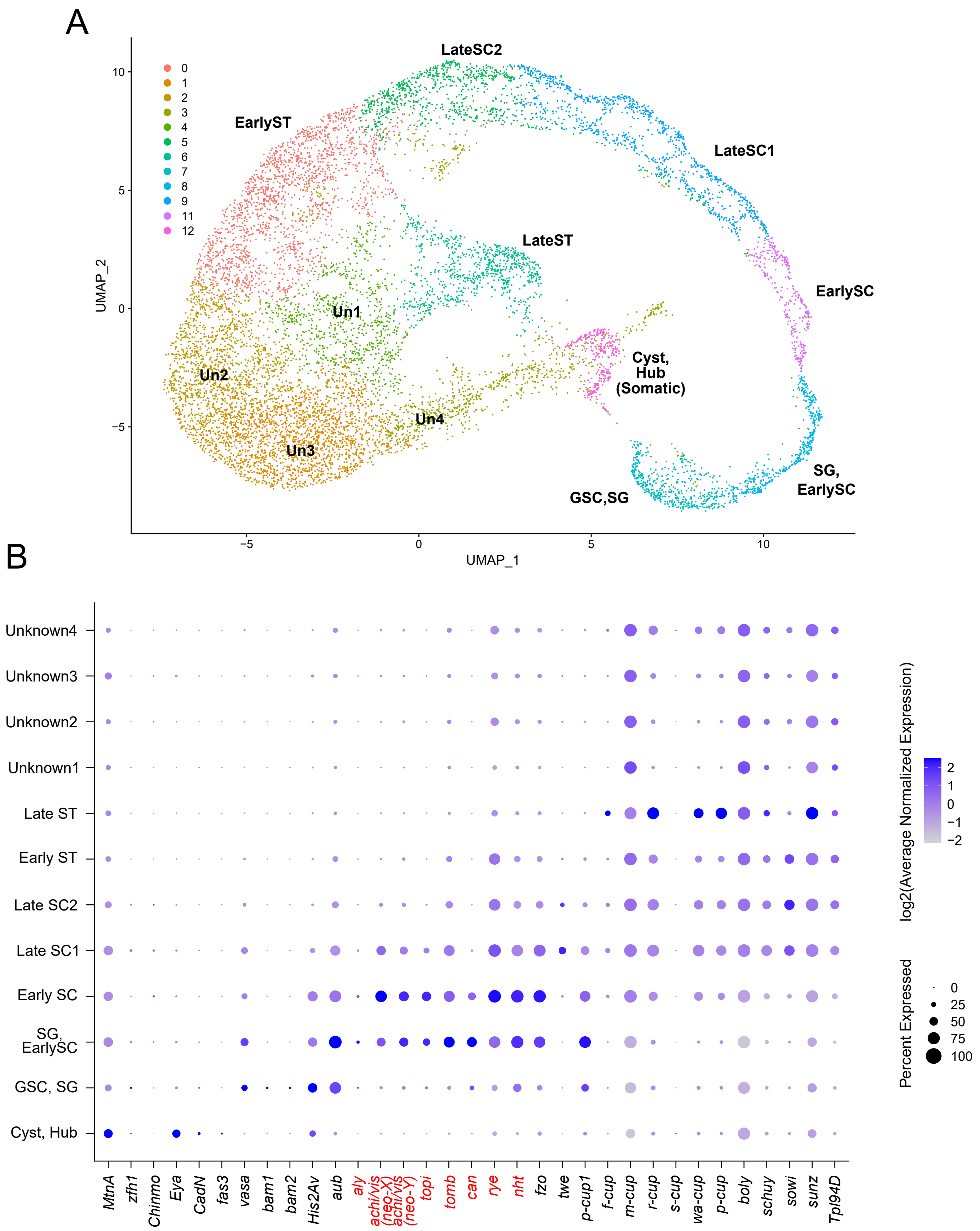

S1 Fig. A. UMAP projection of all UMI clusters. B. Expression of maker genes used to identify cell types across clusters. The group of genes expressed in the spermatocyte necessary for meiosis (collectively known as the meiotic arrest genes) are labeled in red.
