# Supplementary material for: Single-cell RNA-seq of Drosophila miranda testis reveals the evolution and trajectory of germline sex chromosome regulation
Source: PLoS Biol. 2024 Apr 30;22(4):e3002605. doi: 10.1371/journal.pbio.3002605 (PMC11135767; doi:10.1371/journal.pbio.3002605)
Supplement: S2 Fig — * = p < 0.000001, Wilcoxon’s rank sum test. The data underlying this figure can be found in S1 Data. (PDF) [file pbio.3002605.s005.pdf]

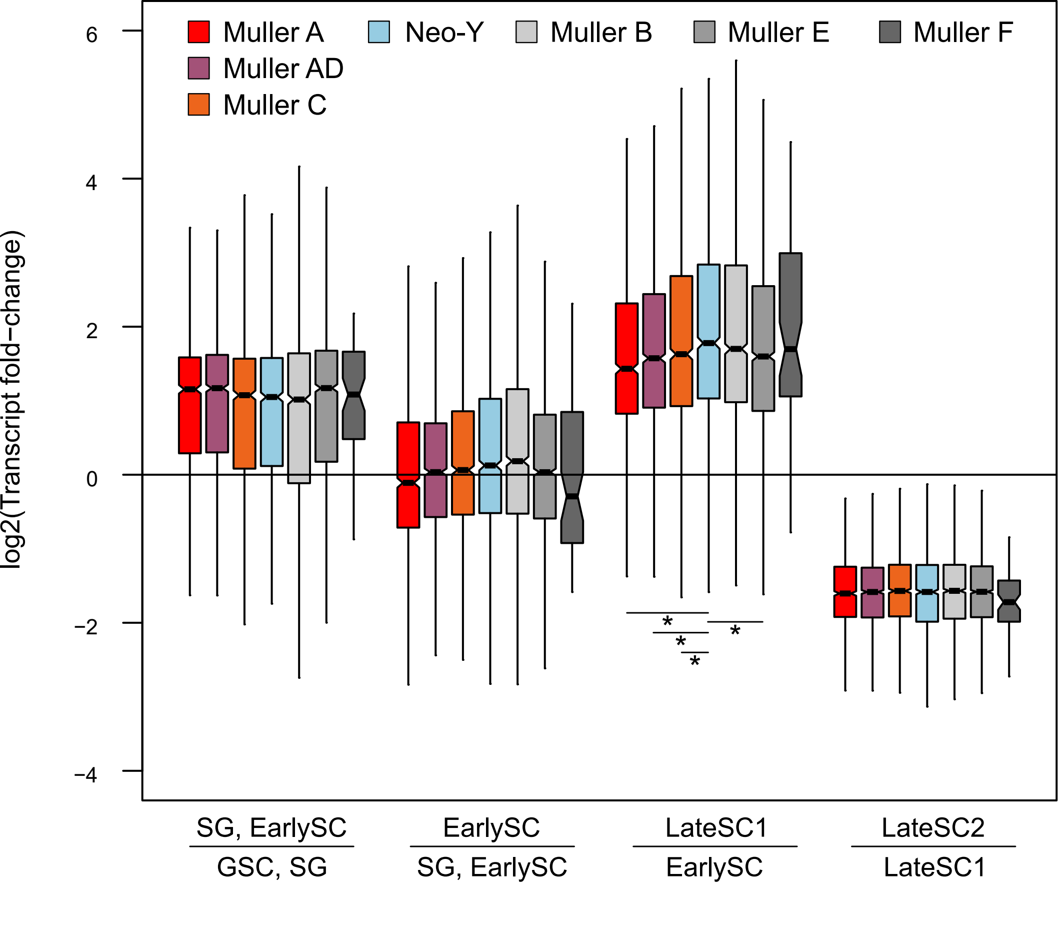

S2 Fig. Log scale distribution of expression fold differences between different cell stages for different chromosomes. * = $p < 0.000001$, Wilcoxon's rank sum test.
