# Supplementary material for: Single-cell RNA-seq of Drosophila miranda testis reveals the evolution and trajectory of germline sex chromosome regulation
Source: PLoS Biol. 2024 Apr 30;22(4):e3002605. doi: 10.1371/journal.pbio.3002605 (PMC11135767; doi:10.1371/journal.pbio.3002605)
Supplement: S3 Fig — (A) By using a more sensitive clustering parameter, additional clusters were identified for the spermatocytes which may correspond to different sperm types. (B) X:A ratio of the sex chromosomes in the subclusters. The data underlying this figure can be found in S1 Data. (PDF) [file pbio.3002605.s006.pdf]

A

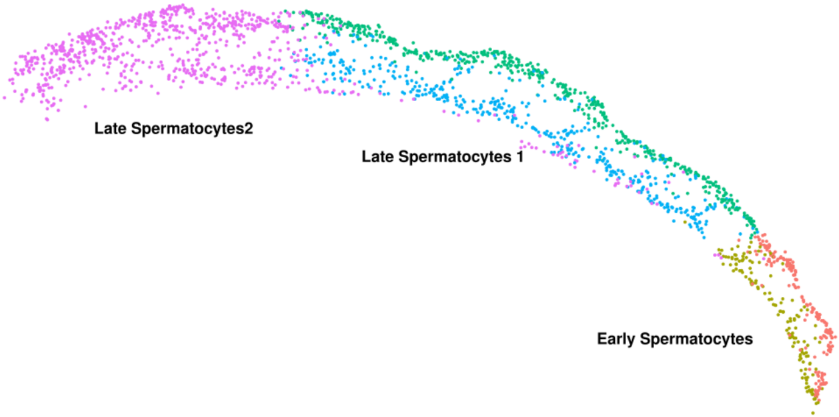

B

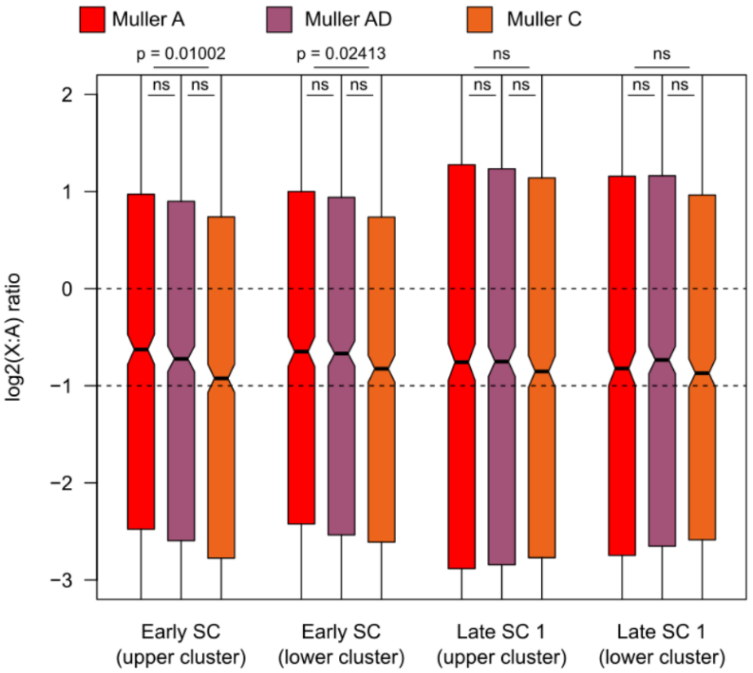

S3 Fig. Subclusterring of spermatocyte stages. A. By using a more sensitive clustering parameter, additional clusters were identified for the spermatocytes which may correspond to different sperm types. B. X:A ratio of the sex chromosomes in the subclusterings.
