# Supplementary material for: Single-cell RNA-seq of Drosophila miranda testis reveals the evolution and trajectory of germline sex chromosome regulation
Source: PLoS Biol. 2024 Apr 30;22(4):e3002605. doi: 10.1371/journal.pbio.3002605 (PMC11135767; doi:10.1371/journal.pbio.3002605)
Supplement: S4 Fig — (A) Testes-expressed genes across meiotic stages: genes in the scRNA-seq data were selected based on bulk testes RNA-seq. Testes-expressed genes are classified as those expressed in the upper 50% of the expression (TPM) distribution. (B) All genes were used if they show more than 10 reads in the scRNA-seq experiments. The data underlying this figure can be found in S1 Data. (PDF) [file pbio.3002605.s007.pdf]

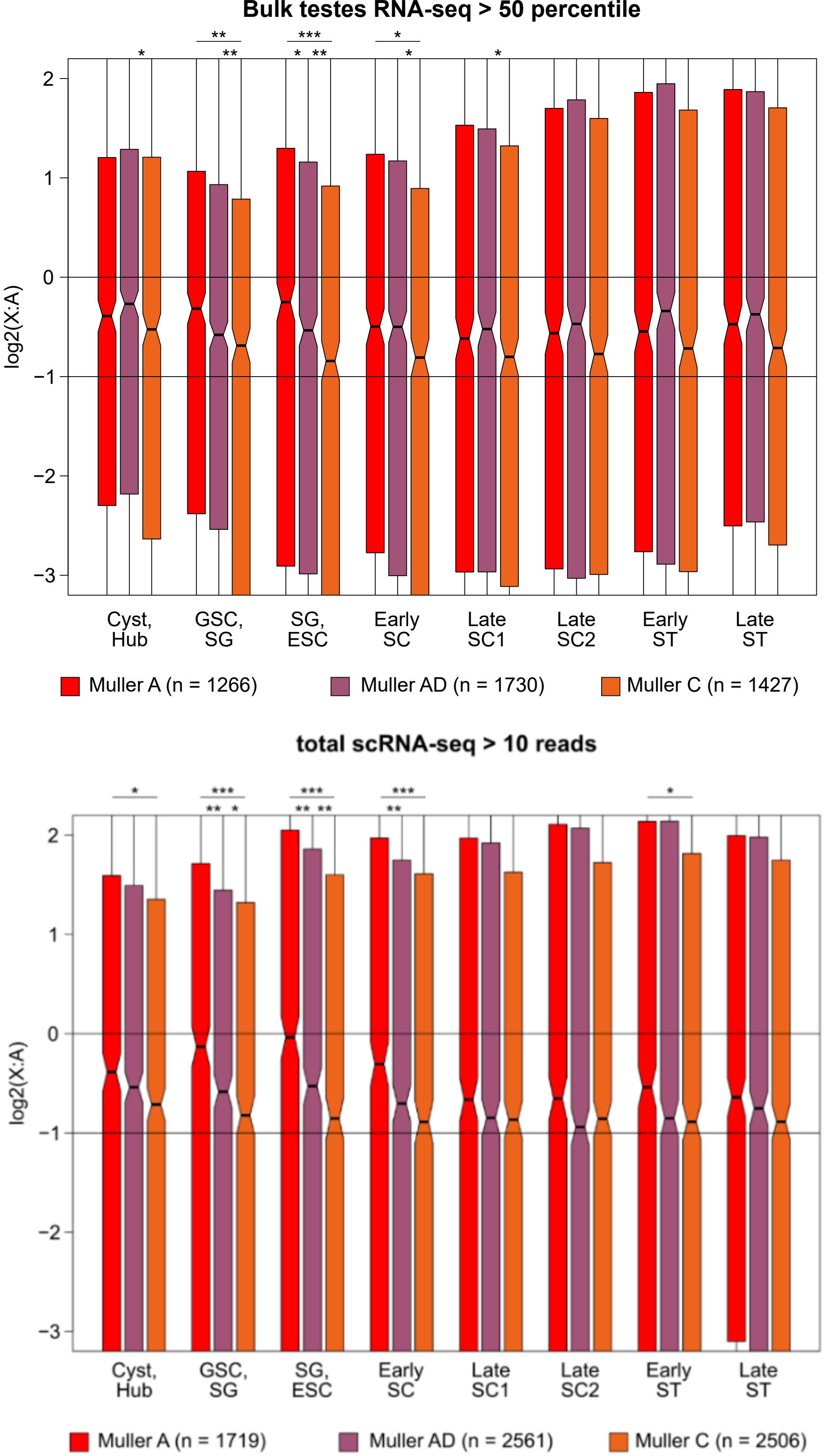

S4 Fig. X:A ratios of different gene sets. A. Testes-expressed genes across meiotic stages: genes in the scRNA-seq data were selected based on bulk testes RNA-seq. Testes-expressed genes are classified as those expressed in the upper 50% of the expression (TPM) distribution. B. All genes were used if they show more than 10 reads in the scRNA-seq experiments.
