# Supplementary material for: Single-cell RNA-seq of Drosophila miranda testis reveals the evolution and trajectory of germline sex chromosome regulation
Source: PLoS Biol. 2024 Apr 30;22(4):e3002605. doi: 10.1371/journal.pbio.3002605 (PMC11135767; doi:10.1371/journal.pbio.3002605)
Supplement: S5 Fig — (A) X:A ratio depending on distance from MSL peaks. (B) Normalized read counts of the Xs depending on distance from MSL in the early testes stages. The data underlying this figure can be found in S1 Data. (PDF) [file pbio.3002605.s008.pdf]

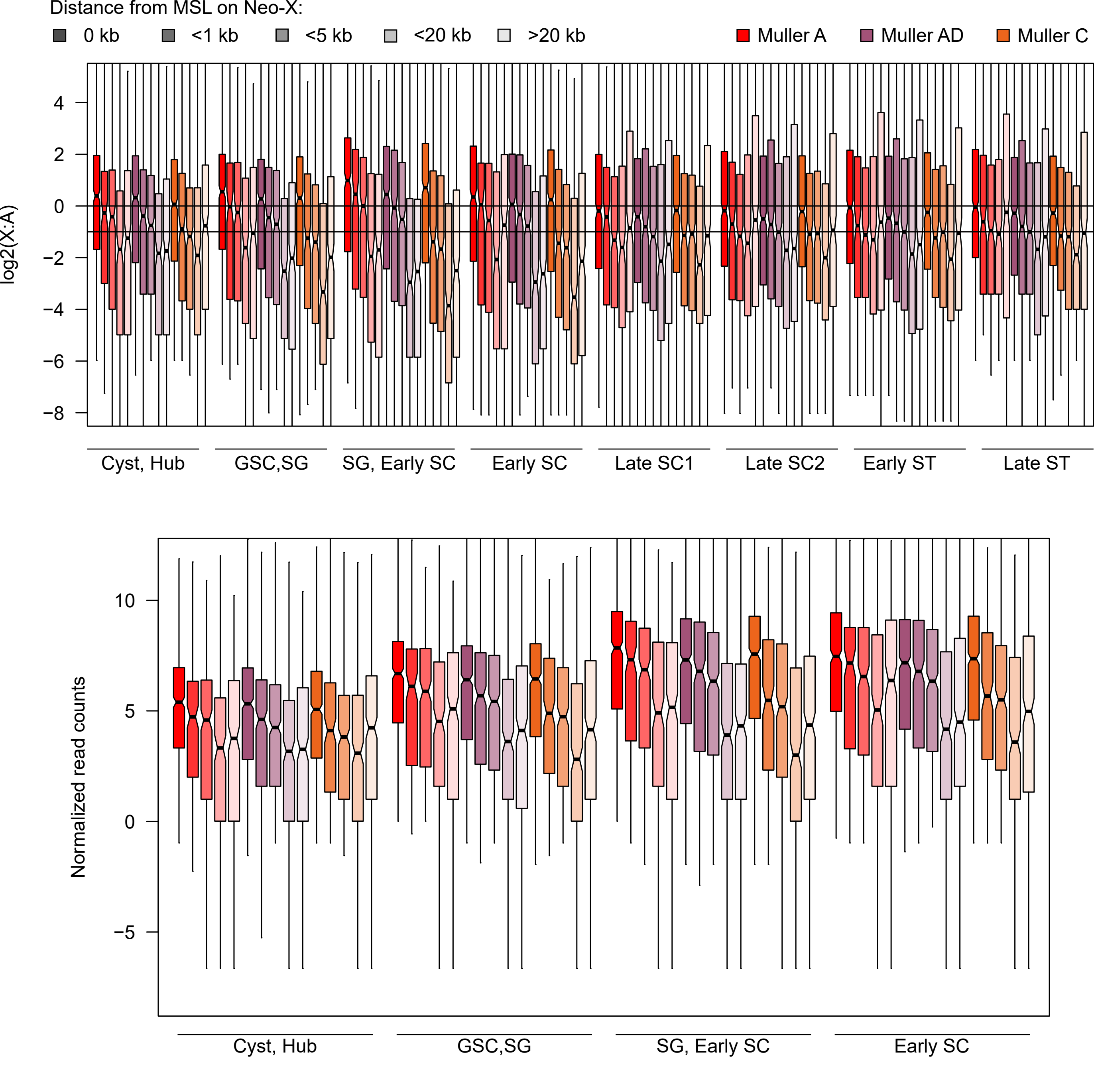

S5 Fig. A. X:A ratio depending on distance from MSL peaks. **B.** Normalized read counts of the Xs depending on distance from MSL in the early testes stages.
