# Supplementary material for: Single-cell RNA-seq of Drosophila miranda testis reveals the evolution and trajectory of germline sex chromosome regulation
Source: PLoS Biol. 2024 Apr 30;22(4):e3002605. doi: 10.1371/journal.pbio.3002605 (PMC11135767; doi:10.1371/journal.pbio.3002605)
Supplement: S6 Fig — Genes are divided based on their distance from MSL peaks (columns 1–4). Across meiotic stages (rows), log2 scale gene expression is normalized by the median autosomal counts, and their distributions are depicted as kernel densities. Column 5 shows the distribution of autosomal genes found on Muller B and E. Vertical lines within plots demarcate X:A ratios of 1. The data underlying this figure can be found in S1 Data. (PDF) [file pbio.3002605.s009.pdf]

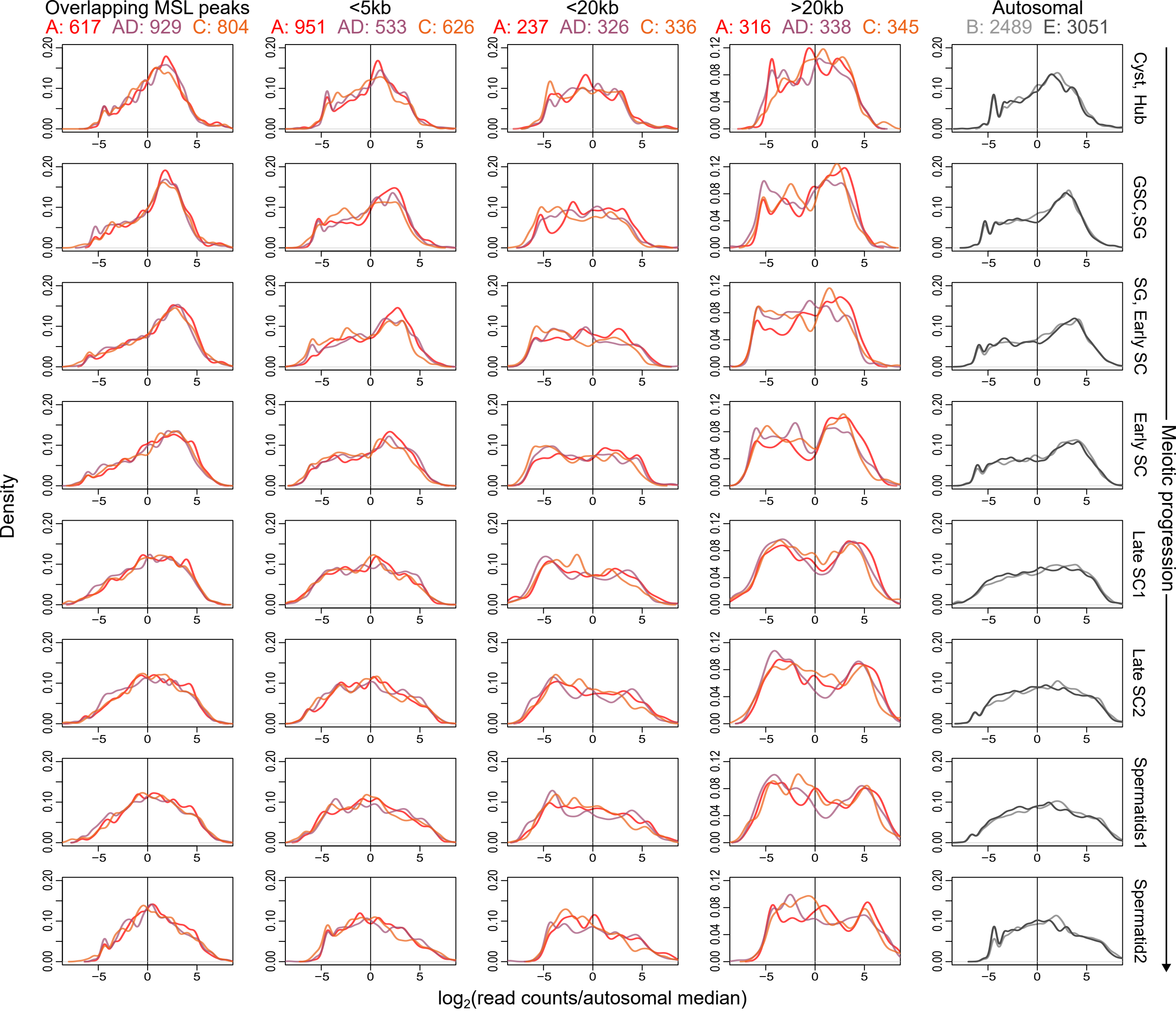

S6 Fig. Distinct transcriptinoal landscape across meiotic progression for genes distal and proximal to MSL. Genes are divided based on their distance from MSL peaks (columns1-4). Across meiotic stages (rows), log2 scale gene expression is normalized by the median autosomal counts, and their distributions are depicted as kernel densities. Column 5 shows the distribution of autosomal genes found on Muller B and E. Vertical lines wihtin plots demarcate X:A ratios of 1.
