# Supplementary material for: Single-cell RNA-seq of Drosophila miranda testis reveals the evolution and trajectory of germline sex chromosome regulation
Source: PLoS Biol. 2024 Apr 30;22(4):e3002605. doi: 10.1371/journal.pbio.3002605 (PMC11135767; doi:10.1371/journal.pbio.3002605)
Supplement: S7 Fig — The data underlying this figure can be found in S1 Data. (PDF) [file pbio.3002605.s010.pdf]

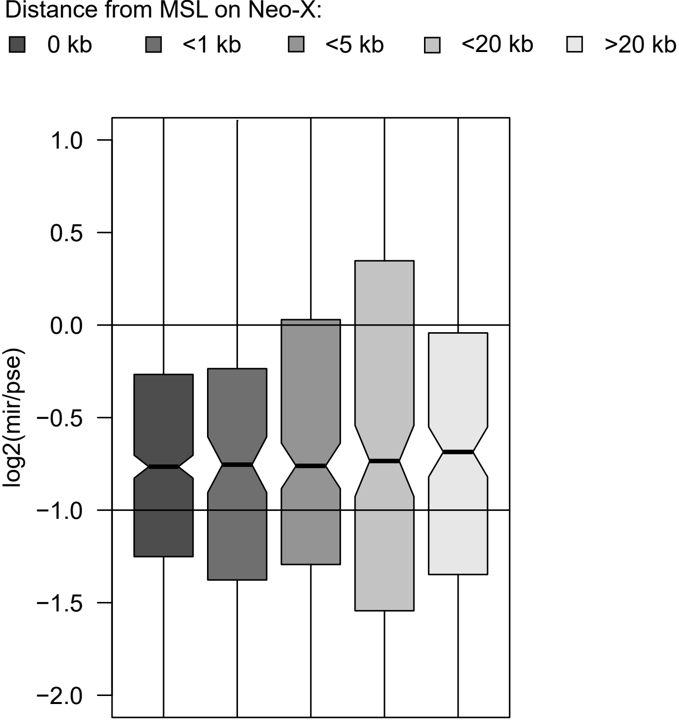

S7 Fig. Testes expression difference between neo-X (*D.miranda*) and autosomal (*D. pseudoobscura*) orthologs.
