# Supplementary figures and images for: Single-cell RNA-seq of Drosophila miranda testis reveals the evolution and trajectory of germline sex chromosome regulation
Source: PLoS Biol. 2024 Apr 30;22(4):e3002605. doi: 10.1371/journal.pbio.3002605 (PMC11135767; doi:10.1371/journal.pbio.3002605)

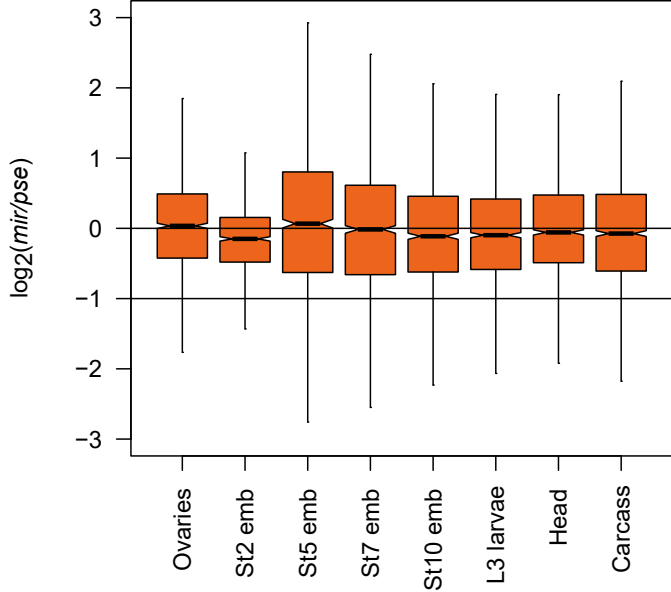

**S8 Fig.** Expression difference between Muller C orthologs in female tissues

Supplement: S8 Fig — The data underlying this figure can be found in S1 Data. (PDF) [file pbio.3002605.s011.pdf]

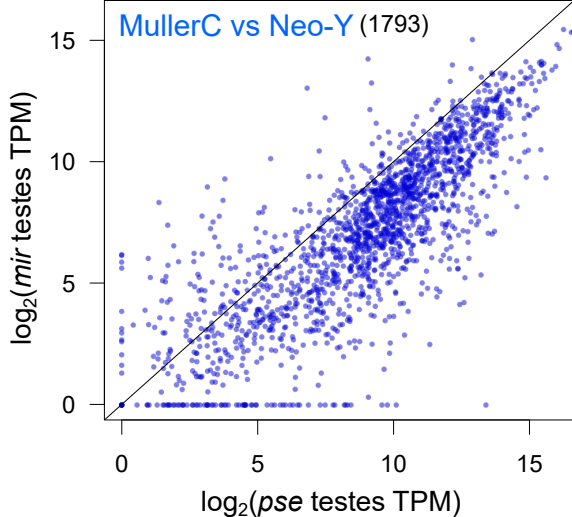

**S9 Fig.** Muller-C expression is *D. pseudoobscura* vs neo-Y expression in *D. miranda*.

Supplement: S9 Fig — The data underlying this figure can be found in S1 Data. (PDF) [file pbio.3002605.s012.pdf]
