# Supplementary material for: Single-cell RNA-seq of Drosophila miranda testis reveals the evolution and trajectory of germline sex chromosome regulation
Source: PLoS Biol. 2024 Apr 30;22(4):e3002605. doi: 10.1371/journal.pbio.3002605 (PMC11135767; doi:10.1371/journal.pbio.3002605)
Supplement: S10 Fig — (A) Ribosome profiling reads in TPM for neo-Y genes with untruncated or truncated CDS. Neo-Y genes are considered truncated if the CDS is shorter than the neo-X CDS by 20%. (B) Ratio of ribosome profiling reads of sex chromosomes to autosomes. (C) Ribosome occupancy as measured by the ratio ribosome profiling reads (in TPM) over RNA-seq reads (in TPM) for the chromosomes. * = p < 0.0001. The data underlying this figure can be found in S1 Data. (PDF) [file pbio.3002605.s013.pdf]

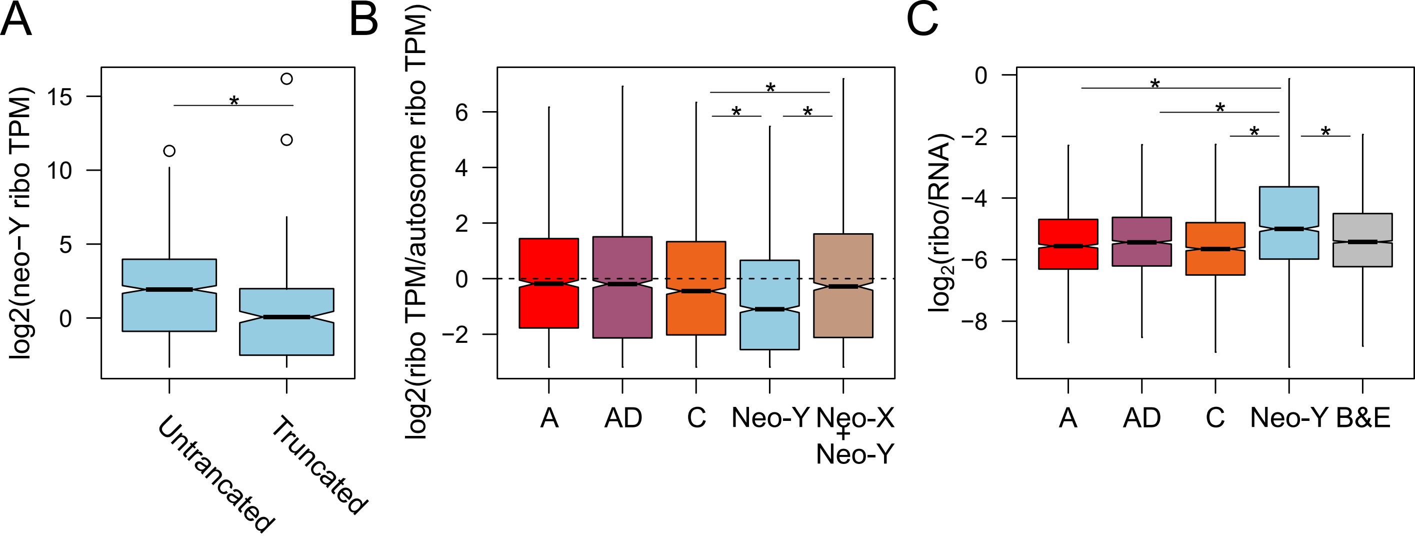

S10 Fig. Ribosome profiling and occupancy of *D. miranda* male larvae. A. ribosome profiling reads in TPM for neo-Y genes with untruncated or truncated CDS. Neo-Y genes are considered truncated if the CDS is shorter than the neo-X CDS by 20%. B. Ratio of ribosome profiling reads of sex chromosomes to autosomes. C. Ribosome occupancy as measured by the ratio ribosome profiling reads (in TPM) over RNA-seq reads (in TPM) for the chromosomes. * = $p < 0.0001$.
