# Supplementary material for: Single-cell RNA-seq of Drosophila miranda testis reveals the evolution and trajectory of germline sex chromosome regulation
Source: PLoS Biol. 2024 Apr 30;22(4):e3002605. doi: 10.1371/journal.pbio.3002605 (PMC11135767; doi:10.1371/journal.pbio.3002605)
Supplement: S11 Fig — Contrast between multicopy genes on the neo-X and neo-Y for copy number (A), abundance of sense smRNA (B), and abundance of antisense smRNA (C). The data underlying this figure can be found in S1 Data. (PDF) [file pbio.3002605.s014.pdf]

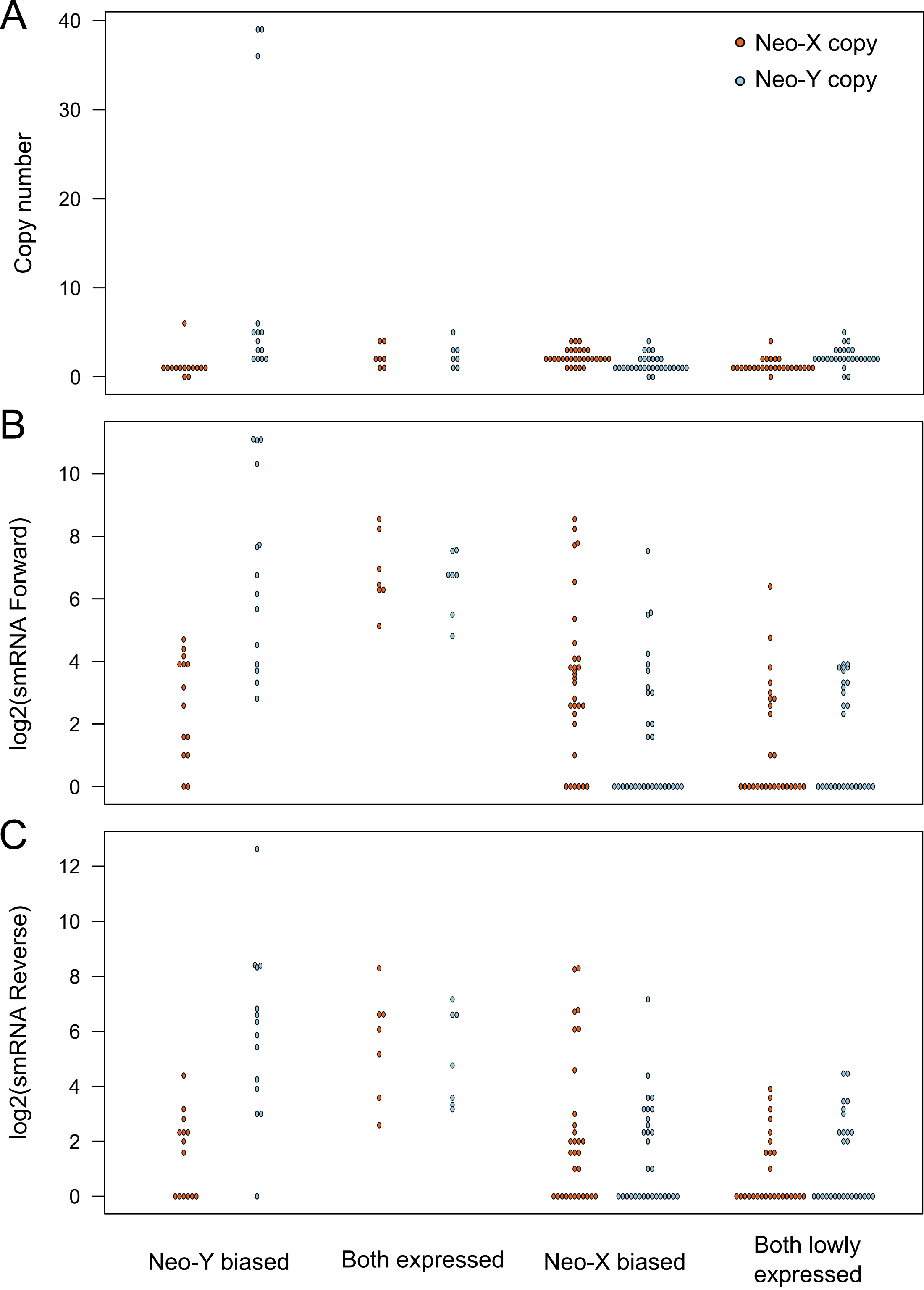

S11 Fig. Contrast between multicopy genes on the neo-X and neo-Y for copy number (**A**), abundance of sense smRNA (**B**), and abundance of antisense smRNA (**C**).
