# Supplementary material for: Single-cell RNA-seq of Drosophila miranda testis reveals the evolution and trajectory of germline sex chromosome regulation
Source: PLoS Biol. 2024 Apr 30;22(4):e3002605. doi: 10.1371/journal.pbio.3002605 (PMC11135767; doi:10.1371/journal.pbio.3002605)
Supplement: S13 Fig — (A) Expression of neo-Y-linked genes across developmental stages and tissue types. * = p < 2.2e-16, Wilcoxon’s rank sum test when compared to testes expression. (B) Expression of Y-specific genes across developmental stages and tissue types in D. pseudoobscura and D. miranda. The data underlying this figure can be found in S1 Data. (PDF) [file pbio.3002605.s016.pdf]

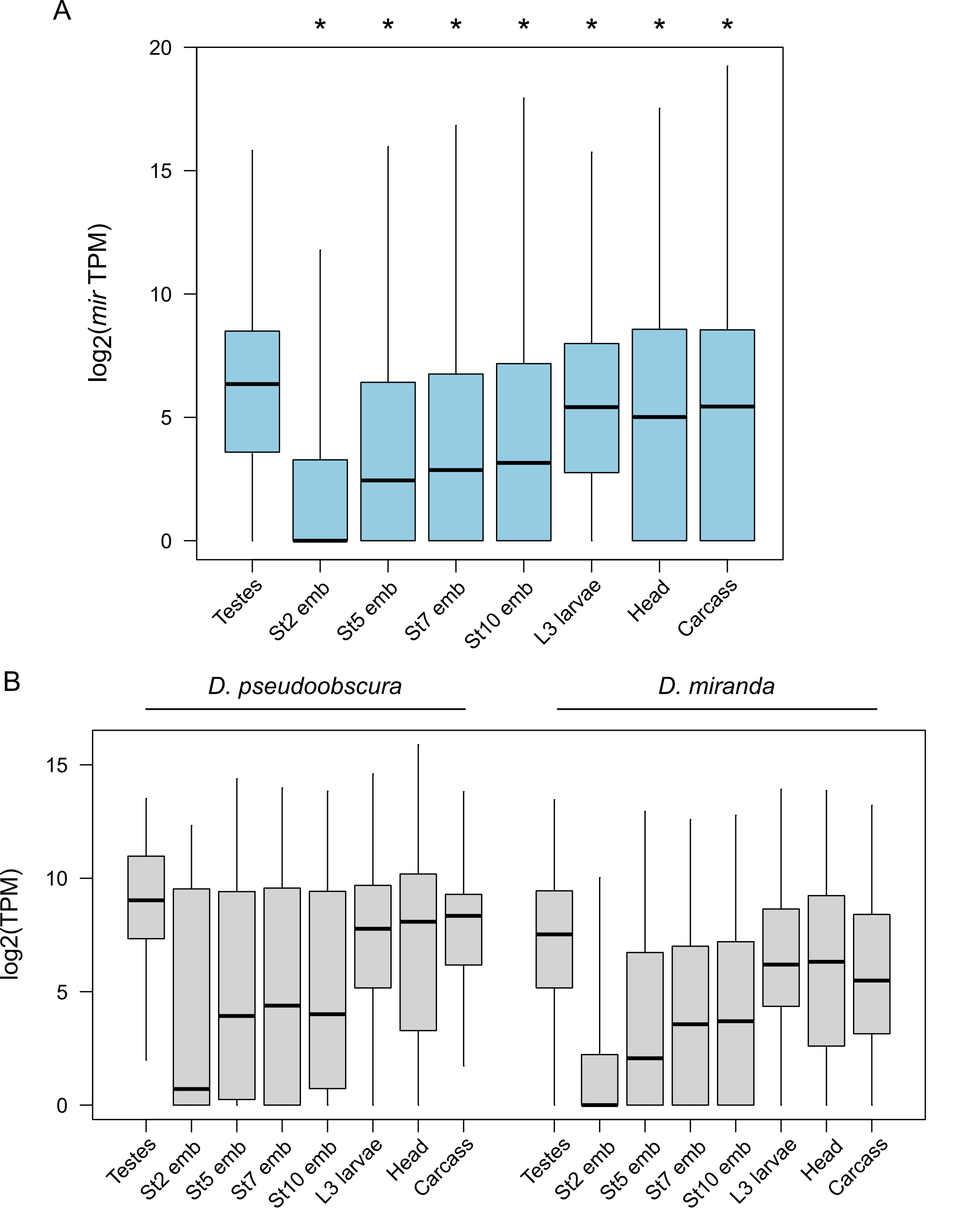

S13 Fig. A. Expression of neo-Y-linked genes across developmental stages and tissue types. * = $p < 2.2e-16$, Wilcoxon's rank sum test when compared to testes expression. **B.** Expression of Y-specific genes across developmental stages and tissue types in *D. pseudoobscura* and *D. miranda*.
