# Supplementary material for: Single-cell RNA-seq of Drosophila miranda testis reveals the evolution and trajectory of germline sex chromosome regulation
Source: PLoS Biol. 2024 Apr 30;22(4):e3002605. doi: 10.1371/journal.pbio.3002605 (PMC11135767; doi:10.1371/journal.pbio.3002605)
Supplement: Attachment — Submitted filename: Reviewer_comments_final.docx [file pbio.3002605.s018.docx]

Dear Editors,

Please find enclosed a substantially reversed version of our manuscript "Single cell RNA-seq in Drosophila testis reveals evolutionary trajectory of sex chromosome regulation". Below is a point-by point response to each reviewer and the associate editor. We hope that you find our revised version suitable for publication in PLoS Biology.

Best wishes

Doris Bachtrog


**Response to Reviewer #1:**

Drosophila miranda is a rich resource for understanding the evolution of X chromosomes, and it may shed light on this chromosome's epigenetic trajectory. However, in my opinion, this study is not appropriate for publication in PLoS Biology because the lack of cytological experiments may lead the authors to draw insufficient or incorrect conclusions. The field of MSCI in Drosophila has already been the subject of lengthy debate, largely due to similar issues that hampered scientific progress. The Sex Chromosome Meiotic Inactivation is recognized as an epigenetic phenomenon with cell detectable consequences, and cytology experiments helped to clear up previous misunderstandings.
Below I explain my evaluation in detail.

We agree that cytology can be very valuable. However, it is our understanding of the literature that cytology has contributed as much (or more) to misunderstandings about the absence/presence of MSCI as it has done to clear them up. For example, Lifschytz & Lindsley reported increased X-condensation in early spermatocytes (in support of MSCI), but the cytological data were subsequently refuted by Kremer et al. Mahadevaraju et al. used cytology to measure active Pol-II signals on the X and concluded that reduced levels of active Pol-II (around half compared to autosomes) supports MSCI, but a genome-wide profiling study found that levels of active Pol-II on the X are fully consistent with lack of dosage compensation, and found no evidence of MSCI (for details, see p. 15-17 in our manuscript).

Major points

1) Cytological experiments are necessary for detecting and classifying cell clusters in Drosophila miranda:

a) In comparison to the majority of the other species in the genre, the Drosophila pseudoobscura group has a distinct testis morphology. The organ's external format is a result of different spermatogenesis development in terms of shape, size, and process. For starters, instead of four mitotic divisions, the species have five and the final product is 128 spermatozoa for each initial cell. Second, two types of spermatozoa are produced. It means that this group is known to have sperm polymegaly, a type of sperm heteromorphism characterized by the production of two different sizes of spermatozoa within the same animal, with the only difference being the lengths of the head and tail. Furthermore, genes expressed in the testes of D. pseudoobscura have been found to be less similar to their D. melanogaster orthologs  (PMID: 15632085).

These are important points that we should have made clear. We have now added text (first section of Results; p. 5) to discuss these morphological differences in the obscura group (5 rounds of mitosis instead of 4 and heteromorphic sperm types). Despite these differences, however, we see the same overall transcriptional trajectory across spermatogenesis as has been found in *D. melanogaster*, with the similar combinations of marker genes coming up in different cell types. This strongly supports that overall regulatory programs of spermatogenesis are conserved across *Drosophila*.

b) As a result, genetic markers with known expression in different testis cell phases of Drosophila melanogaster should not be used for definitive cluster classification. In the absence of a Drosophila miranda testis expression marker, authors should rely on genes that are only expressed in specific clusters for in situ hybridization confirmation of their predictions.

Because there is currently no RNA in situ data in *D. miranda* or *D. pseudoobscura*, this unfortunately cannot be done. While there are undoubtedly differences in the expression of individual germline testes genes, we note that the trajectory of expression of the marker genes show broad and remarkable similarities between *D. melanogaster* and *D. miranda* (for example, *vasa* being highly expressed in early spermatogenesis and the *cup* genes which are required form spermiogenesis being expressed toward the later stages). Moreover, the key stages of interest for the topic of meiotic regulation are the spermatocytes/meiotic stages. We have further increased the number of marker genes for these stages by adding genes necessary for meiosis in the spermatocytes (known as the meiotic arrest genes, which are known to be transcribed starting in the primary spermatocytes; see Cooper-White 2010) in Supplementary Figure 1B. Their expression pattern is clearly limited to the cells we labeled as spermatocytes. We observed the expected trajectory and the expected expression pattern of most of the marker genes from *D. melanogaster*, showing that there are clearly enough similarities between the species to make this interspecies inference meaningful. Thus, it seems very unlikely that transcriptional regulation is wildly different between *D. melanogaster* and *D. miranda* spermatogenesis.

c) Clusterization and confirmation are not working properly with the author's current method, as shown in Figure 1 and Supplementary Figure 1. First, there are four unknown clusters with strikingly similar expression profiles. Second, the progression from late SC1 to ST exhibits the same problem. There is no evidence of polymegaly progression. As similar patterns of expression can lead to misclassification and thus further misinterpretation about timing and specific level and stage of X chromosome expression regulation, cytological characterization could shed light on these issues.

There is currently no understanding of the developmental and/or genetic basis of these two sperm types. Therefore, expectation of clear clustering between the types are unwarranted. Nevertheless, we believe our clustering actually shows some evidence of eusperm and parasperm. In the UMAP figure (1E), there are two different groups (parallel streaks) within the early and late spermatocyte clusters, which can be distinguished using a more sensitive clustering parameters. Para- and/or eusperm may also account for the unidentified cell types which - as we have mentioned now - likely correspond to more mature sperm types.

We have now included this figure as **Supp figure 3A**, but noted the speculative nature of this inference. Extensive experiments and studies (likely encompassing entire if not multiple research programs) are needed to characterize the genetic and developmental bases of heteromorphic sperm types, which are required for us to conclude whether these clustering results correspond to heteromorphic sperm types. While we are interested in pursuing such inquiries, they are way beyond the scope of the current manuscript.

Moreover, for the purpose of this study and our inference regarding X regulation, even if we separate out the spermatocyte stages into smaller clusters, we still see the same pattern of expression on the different aged-sex chromosomes consistent with our interpretation of the loss of DC across meiosis, whereby the three X’s equalize across meiotic progression with the X:A ratio remaining above and closer to 0.5 (now included as **Supp fig. 3B**).

For the unidentified clusters (which may be different types of mature spermatids), all three Xs show similar expression levels much like other post-meiotic stages.

2) Cytological experiments are needed to differentiate between mRNA level and transcription

a) Because cells have a fixed rate of RNA degradation, mRNA expression measurements are a proxy for transcription. However, drosophila spermatogenesis is known for producing a large number of proteins that are required later in the post-meiotic stages and are translated from mRNAs produced during meiosis and stored in the cytoplasm (Olivieri and Olivieri 1965, Schäfer et al 1995). That is why the proportion of X/A should not be used alone to distinguish between MSCI and a lack of dosage compensation. mRNAs transcribed in previous stages are stored inside cells during later stages of development, which may prevent mRNA sequence methods from detecting transcriptional shut off. Cytological experiments have already been performed in Mahadevaraju S. et al, 2021, demonstrating the cytological absence of active RNA polymerase on the X chromosome territory in D. melanogaster spermatocytes and are a promising resource to solve this problem D. miranda.

We agree that mRNA level is not the same as transcription rate and that lingering transcripts produced from previous stages can temper the extent of down regulation. The expectation from this scenario is that X-linked transcript abundances as measured by RNA-seq decreases slower than actual inactivation. And importantly, when comparing the three different Xs, the neo-X which would have incomplete MSCI due to its young age should show transcript abundance higher than the older Xs but lower than the autosomes.

However, these expectations are inconsistent with our observations: First the overall transcript abundance actually increases in the spermatocyte stages (also observed by Witt et al, particularly at early spermatocytes; unclear from Mahadevaraju et al as only X:A is reported without reporting expression levels of the X). Second, more importantly, when comparing between the Xs, expression or transcript abundance of the old (Muller-A) and intermediate (Muller-D) X arms are higher prior to meiosis and equalize with the neo-X in late spermatocytes at a level approaching half the expression of the autosomes.

Regarding the cytological experiment by Mahadevaraju et al. First, Mahadevaraju et al. did not show that active Pol-II is absent on the X, but instead they report that the ratio of active to total Pol-II is decreased on the X relative to autosomes (their Fig 6). Importantly, they also used cytology to show that the volume of the X is actually significantly greater than that of a similarly sized autosomal region (see their Fig 5i and their paragraph about X chromosome condensation in primary spermatocytes). This cytological data is opposite to what is expected under MSCI but fully consistent with a model of dosage compensation shutoff during meiosis. DC in early spermatogenesis would increase the volume of the X (as observed by Mahadevaraju et al), and its shutoff could result in active Pol-II levels to be reduced on the X.

We agree that the reduced Pol-II activity shows reduced expression on the X. However, we disagree that the results can unambiguously resolve two types of down regulation MSCI or loss of DC. The immunostaining of phosphorylated Pol-II appeared to show lower Pol-II initiation (Pol-II serine 5 phosporylation) and elongation activity (Pol II serine 2 phosphorylation) in the X chromosome territories compared to autosomal territories (Figure 6 in Mahadevaraju et al). The reported values are ratios to all Pol II and for both active marks are around half. This is to say that only half of the Pol II in X territories are active which is well within the range of DC loss. In contrast, the active pol II to all pol II ratios are closer to 1. Further the loss of phosophoserine 5 and 2, which represent initiating and elongating pol II, is consistent with the loss of DC, since DC in Drosophila is characterized by increased Pol II pausing at the 5’ right after the TSS and increased elongation rate (Conrad et al. 2012; Francesco Ferrari et al. 2013) – although we note that whether DC involves more initiation is contentious (F. Ferrari et al. 2013; Straub and Becker 2013; Vaquerizas et al. 2013). This discussion is now added to the first section of the Discussions.

Importantly, a recent genome-wide study profiling active Pol-II across the genome also concluded that the pattern of chromosomal active Pol-II is fully consistent with a shutoff of dosage compensation in spermatocytes. Anderson et al. found increased levels of active Pol-II on the X in wings (somatic tissue with dosage compensation), but similar levels of active Pol-II on the X and autosomes in spermatocytes (Fig 4 in Anderson et al). That study also reported a lack of silencing marks on the X, and concluded that there is a shutoff of dosage compensation during spermatogenesis, but no evidence for MSCI in flies.

b) Also, it is not possible to use Inference of transcriptional kinetics parameters as done by the authors. In the model of transcription used, it is assumed that a gene switches to the on state and off state at exponentially distributed times with rates, and, regardless of gene state, the RNA is degraded with fixed rate which is not true for drosophila spermatogenesis.

We have removed the burst rate analyses.

c) The authors have mentioned in the discussion:
   "Discrepancies have been attributed to limitations in methodologies including limited number of genes quantified and tissue contamination in manual dissections, and single cell RNA-seq should be able to resolve the debate. Analyzing the meiotic cell types in adult Drosophila testes scRNA-seq, Witt et al found no evidence of MSCI [35]. However Mahadevaraju et al reported the presence of MSCI after analyzing scRNA-seq generated from developing testis discs in male larva [36]. Therefore, even with scRNA-seq the status of MSCI in Drosophila remains unclear."

  First, it is a misinterpretation to think that Witt et al. and Mahadevaraju et al. came to a different conclusion. The first group found Dosage compensation in mitotic cells and the second group found the same results and also MSCI in meiotic cells. The former results were based on cytological evidence of lack of active RNA polymerase on the X in spermatocytes. Second, if, even scRNA-seq was not able to solve the MSCI debate in Drosophila (because of the reasons explained in 2A), why do the authors think that the same technique will shed a light on MSCI status in D. miranda? Third, it is surprising that an important work such as the one done with cytological experiments is only mentioned for the first time in this study's discussion.

Witt et al state “Our results do not provide evidence to support or disprove MSCI in late germ cells. We observed, in spermatocytes and spermatids, that X chromosome genes are expressed with median RNA counts of 63–77 percent that of autosomal genes. This would be expected from incomplete or absent dosage compensation, as some transcripts present in these cells would be retained from earlier stages despite reduced X transcription. For us to confidently predict the presence of MSCI, we would have to observe X:autosome ratios <0.5 in meiotic and postmeiotic germ cells.” While the authors suggest they cannot disprove MSCI, their following verbiage implies that incomplete or absence of dosage compensation is more consistent with their data. We have rephrased our text to capture the noncommittal wording. However, as astutely pointed out by Witt et al, an X:autosome ratio < 0.5 is needed to confidently confirm MSCI and rule out absence of DC. As far as we can tell, neither Witt et al nor Mahadevaraju et al (or our study) showed reduction of expression that meets this criterium. Also see our answer above to question the conclusions of Mahadevaraju based on their cytological data.

We do not claim that another scRNA-seq study is key for settling this debate. The key advance in our study is to use the unique sex chromosome architecture of *D.* miranda to add an evolutionary and comparative framework in addressing this issue. Given that neo-sex chromosomes must gradually evolve characteristics of bona-fide/old sex chromosomes, such as dosage compensation on the X, it stands to reason that MSCI also needs to evolve, hence our model to differentiate between the possibilities (MSCI vs Loss of DC). This can only be done when there are multiple X-chromosomes of different ages within the same meiotic cells which is the case for *D.* miranda. Furthermore, the presence of the autosomal Muller C in *D. pseudoobscura* provides an unambiguous point of comparison to determine whether the X and subsets of genes on the X are specifically silenced. By analyzing the orthologs and gametologs which we have added as Figure 3 B and C, we show that low expression of certain gene sets are more consistent with ancestrally low expression and not acquisition of X-silencing.

3) MSCI vs. lack of Dosage compensation

a) If the authors had acknowledged previous cytological studies, they could have avoided making assumptions about elaborating their hypothesis. According to the authors, MSCI would predict a positive correlation between age and MSCI level in terms of X chromosome evolutionary history (Figure 1A). Based on cytological data, Mahadevaraju S. et al, 2021 proposed a territory-regulation model for X inactivation that could result in the same level of down regulation of all three different aged chromosomes if they were located in the same territory.

Again, we would like to point out that Mahadevaraju et al. actually showed that the X chromosome shows higher volume than the autosomes. This is not what is expected if certain chromosomal territories are compacted and inactivated.

We also do not agree with the idea of “same level” of down regulation of evolving neo-Xs. First, this is not supported by our data as the neo-X reaches the lowest expression level first, which makes intuitive sense if dosage compensation is less robust on the evolving chromosome.

Second, given that neo-sex chromosomes need to evolve sex-chromosome-specific characteristics (X dosage compensation and Y degeneration), it is a reasonable assumption that MSCI, if it is a conserved chromosomal mechanism, would also need to evolve gradually and hence the a priori expectation is that the neo-X would be at an intermediary state between the X and autosomes. This seems to be less presumptuous than the alternative that the neo-X would be already fully incorporated into the X-territory – requiring the rate of MSCI evolution to be much faster than loss of Y-linked genes or dosage compensation of the X, both of which are incomplete on the neo-Y and X, respectively, (shown by decades worth of papers). Crucially, the neo-X is not physically linked to the ancestral X’s, so it is even more implausible of how this chromosome would be incorporated into such a ‘chromosomal territory’.

Third, if we extend this model of rapid incorporation into inactivating territory to the early stages of neo-sex chromosome evolution, the model seems rather untenable; young neo-X chromosomes would never survive because linkage to the X would lead to rapid silencing of previously autosomal genes. In the case of *D. albomicans*, a >50Mb (>20% of the genome) autosome (which itself is a fusion of Muller C and D) became X-linked through a Robertsonian fusion with Muller A. Unlike Muller C in *D. miranda* which is a separate chromosome from the older X, this fused neo-X would be more likely to be incorporated into the X-territory. However, it seems very unlikely that meiosis will still proceed properly when more than 1/3 of the genome is experiencing a major dosage imbalance through meiosis if the neo-X is silenced and only the neo-Y gametolog can be expressed. We also specifically tested this by looking at neo-X and neo-Y allele specific expression from available testes RNA-seq in testis of this species (Zhou et al 2012 and Wei & Bachtrog 2019) which actually show a slight neo-X bias. This expression data of testis is indicative of early gene-by-gene degeneration of the neo-Y (see Wei & Bachtrog 2019), and is inconsistent with lower neo-X expression as predicted by rapid incorporation into the suppressive territory.

Therefore, we do not think this model of rapid incorporation into inactivating territory (as opposed to gradual like all other sex chromosome characteristics) is viable or supported at all by data.


b) There is a lot of information that can be extracted from Figure 1.
b.1) Data normalization is critical in rna-seq experiments. As a result, using different groups of genes would aid in determining how robust the result interpretation is. For example, counting genes that are normally not or are only weakly expressed in the testis will reduce the average and median expression of each chromosome. As a result, you will most likely underestimate the denominator (autosomal expression), and your X/A ratio will be overestimated.

The X:A ratios are internally controlled for library size normalization factor as the same normalization factor will be used for the numerator and denominator, therefore canceling out. However, what genes to exclude/include is critical and will affect the ratios. But this also comes with trade-offs. On the one hand, we can remove lowly expressed genes as they are not expressed in testes, but this will inflate X:A ratio because the numerator will be over-estimated due to the removal of lowly expressed X-linked genes (either due to X-inactivation or loss of DC) below the cutoff (therefore tempering the downregulation). This seems problematic to us when the X is down-regulated. On the other-hand, if we take an agnostic approach using all genes, we may be including genes that are unrelated to testes as mentioned. This would affect the ratio depending on whether the X or the A are disproportionately represented with testes genes. In the case that the X is under-represented for testes genes, there will be more lowly expressed X-linked genes in testes samples. This should disproportionally deflate the numerator, therefore lowering the ratio. Our current strategy is to use a low read count cut off, removing any gene with <10 total reads. We elected this cutoff to error on the side of less restrictive, which should yield lower X:A ratio, and therefore making it more likely to observe values consistent with inactivation.

We have added an additional filtering strategy (included in in **Sup. figure 4**) using only genes whose expression is at the upper 50 percentile of an independent testes dataset (bulk D.miranda testes RNA-seq), representing testes expressed genes:

As expected, this fiterling leads to slightly more elevated X:A. Crucially, however, the neo-X remains the lowest and all chromosomes remain above X:A of 0.5. Thus, our interpretations are robust to different cut-off values.

b.2) The use of MSL-distal and proximal regions is an intriguing approach, but the data can be explained by factors other than a lack of dosage compensation for X down regulation in meiosis. Male-biased genes, for example, are known to be significantly further away from a HAS than female- and unbiased genes. Male biased genes are typically enriched in testis-biased genes derived from meiotic expression, which is where the transcription burst occurs. As a result, the genes most affected by inactivation may be those MSL-distal simply because they are expressed more in the testis, as shown in Figure 2E, rather than due to a lack of dosage compensation. It is important to note that the MSL distal and proximal regions were created using Chip-seq data that were made with other tissue than testis.

We have addressed this issue now by more closely examining the relationship between proximity to DC and gene expression; the results are in the new section “Distinct germline transcriptional trajectories depending on MSL proximity” and corresponding figures 3A, and supplementary figure 5 and 6. Unlike what is suggested, we find that genes most distal from DC actually include a large fraction of meiotically upregulated genes, contrary to the expectation of inactivation of MSL-distal genes. Meiotically expressed genes appear to be further away from MSL peaks, and this is the case for all three X chromosome arms. This is expected since the MSL complex recognizes actively transcribed genes, and DC is shut off in meiosis. This result is thus consistent with the under-representation of testes and male bias genes around MSL sites.

b.3) Following the authors' logic that Muller C has incomplete dosage compensation in general, and given that MSL-distal regions have the lowest dosage compensation hyper transcription effects, genes in Muller C - MSL-distal could be the best control to test MSCI vs. lack of dosage compensation. Figure 2E shows that in early SC, the median expression of genes in Muller C - MSL-distal is about 10% of autosomal median expression. This appears to be due to a stronger downregulation, such as MSCI, rather than a simple lack of dosage compensation.

Upon finer breakdown of genes by their proximity to MSL, we find that the X:A reduction is the result of a couple of confounding effects which we presented in the new section “Low meiotic expression of a subset of neo-X genes reflects ancestral expression.” First because the autosomal median is going up, many of the lowly expressed genes which tend to be far from MSL have decreasing X:A ratios despite the absolute expression being similar (Supplementary figure 5B). Second, we looked at the expression of genes at various distance to MSL, and compared their expression to the autosomal orthologs and Y-linked gametologs, and find that MSL-distal and lowly expressed genes were ancestrally lowly expressed (Figure 3B and C), instead of being specifically targeted by a suppressive mechanism. Thus, there is no evidence of inactivation of X-linked genes.

b.4) Furthermore, if meiotic cells are losing dosage compensation, why does the level of expression of MSL-distal genes increase from late SC on? Perhaps it is transitioning from X inactivation to a non-dose compensated level. It is important to note that in a model where X inactivation occurs, dosage compensation should not be in place.

With our new/refined analyses, we show that this is primarily driven by increase in expression of a group of meiotically upregulated genes on all X chromosomes that tend to be MSL-distal (Figure 3A, Supplementary figure 5).

4)The sessions "Neo-Y gametologs mitigate neo-X dosage imbalance" and "Neo-Y as an environment to resolve sexual conflict" are the most interesting of the study, but there are some issues.

a) The findings that NeoX+NeoY restores equality with autosomal ratio is interesting. However, for all tissues, these values are greater than one. Indeed, Y linked genes in Drosophila melanogaster (as opposed to other species) have a later peak of expression (meiosis and post-meiosis) in male meiosis due to a chromatin decondensation loop. D. miranda isn't particularly interesting because of its Neo-X and Neo-Y chromosomes. D. pseudoobscura species have a different Y chromosome than D. melanogaster (older than neo X, the YD). Furthermore, ancestral Y single copy genes were transposed to a region that is now fused to the muller F gene (the dot chromosome). They should look at expression of genes in YD and ancestral Y-linked genes to test the hypothesis that neo-Y linked expression may mitigate dosage imbalance from incomplete dosage compensation or loss in the testes.

We are not quite sure what the issue is here. While perhaps not interesting to this particular reviewer, we have not seen the mitigating effect of the neo-Y empirically tested previously. In fact, the importance of having a functional Y-gametolog was recently theoretically derived and shown to affect the efficacy of selection on young X chromosomes (Mrnjavac et al 2023. Evol. Lett). It is not clear whether the described mechanism of Y-looping is occurring on neo-Y in D. miranda. Part of the Y-looping is related to the giant satellite-rich introns of Y-linked genes. Such intron structures are not found on the neo-Y. For older X-chromosomes, how can Y-linked alleles mitigate X-linked dosage issues, when for most of the genes there are only X alleles with the Y version lost to degeneration?

b)

"Previous studies have revealed an excess of genes with testis-expression migrating off the X chromosome in Drosophila [72], and MSCI was invoked to explain this exodus. To determine whether a similar migration of X-linked testes genes to autosomes occurred on the neo-X of D. miranda, we identified orthologs between D. miranda and its sister species D. pseudoobscura where Muller C remains autosomal. Interestingly, we do not find an excess of Muller C genes to have moved to autosomes, albeit the number of gene movements is small (Figure 4A). Interestingly, we find that Muller A appears to be
the most frequent destination for genes previously on the Muller C, which is inconsistent with the need to escape X inactivation. The unique opportunity to resolve sexual antagonism provided by the neo-Y offers an alternative path for genes with testes function to escape the X: After an autosome becomes sex-linked forming neo-sex chromosomes, male-beneficial female-deleterious genes are lost on the X to become Y-exclusive. However, this Y-linkage is only a temporary solution as gene corrosion and regulatory interference and epigenetic conflict from transposable elements will eventually drive these genes to either migrate off the Y or be lost entirely."
This hypothesis is interesting but the data is insufficient to support it. Muller A+AD does not appear to have a bias of Muller C exodus when combined. Please provide statistics and your thoughts.

Our model is that neo-X will lose testes genes that are likely sexually antagonistic, while the neo-Y will retain them. This is supported by the disproportionate number of neo-Y-enriched and neo-Y-only genes that previously have testes-biased expression on autosomes (in D. pseudoobscura). We make no claim that they are more likely to end up on the other X chromosome arms, but merely pointing out that within a very small number of genes that moved chromosomes, neo-X gene movement is inconsistent with MSCI-driven gene movement off X chromosomes. The P-value is added now, and unsurprisingly, not significant.

5) Session "Co-amplified neo-sex genes are predominantly expressed during meiotic stages of spermatogenesis" is hard to review since Figure 6 legend is incomplete.

We have made the legend more descriptive.

Other points:
 1) Figure legends could be improved and made more detailed in general.

Updated.

 2)There are no additional tables available. Raw sc-RNA data are complicated, so meta analyses are useful.

We improved our figure legends, and add more detail.

We also added **supplementary table 1, 2 and 3**, accounting for the number of reads from each chromosome in each cell stage, the number of genes used after filtering strategies, and the number of genes in each MSL-proximity category, respectively.

 3)Materials and methods are skimmed over. I couldn't find any additional materials or methods.

We have updated the materials and methods section, especially clarifying how cell-type specific and chromosome specific gene expression and X:A ratios are obtained.

**Response to Reviewer #2:**

In this manuscript, Wei et al. examined the evolutionary trajectory of sex chromosome regulation in Drosophila testis through single-cell RNA-seq analysis. One major question in Drosophila testis biology is whether dosage compensation and  MSCI exist in pre-meiotic germ cells. The authors used D. miranda, a species with recent chromosome fusions, to study this question. They found that dosage compensation exists, a pattern that is consistent with Witt et al. PLOS Genetics 2021 (PMID: 34403408). Compared to Witt et al., they further showed incomplete dosage compensation at various muller elements. The sex chromosomes exhibit a gradual transition in gene regulation patterns during evolution, leading to an increase in the number of genes expressed in the X chromosome in somatic and germ cells. Their results do not support MSCI in D. miranda, further suggesting that there is a need to re-examine the extent to which MSCI exists in Drosophila. The manuscript is\overall well written, although there were quite a few errors in the bibliography (probably caused by a software glitch) that made it hard to evaluate some of the conclusions. The conclusions are scientifically sound, and I have a few questions.

1) If there a revision of the manuscript, please add line numbers.

Added

2) Figure 1F, compared to this work, there was much more decreasing expression in spermatids compared to spermatocytes from previously reported work in D. melanogaster. What would be the possible explanations? Do you think D. miranda specific biology plays a role?

In Witt et al 2021 Figure 2, expression ( Log2(counts per gene) ) in late spermatocytes to late spermatids decrease from ~8 to ~4. D. miranda shows a pretty similar decrease with log2(normalized read counts) of ~9 (later spermatocytes), ~7,5 (early spermatids), ~5 (early spermatids). Note, we do have an additional late spermatocyte stage. So, the scale of expression changes in the two studies are actually quite similar showing a reduction of ~16-fold.

3) "The X chromosomes arms consistently show lower expression than the autosomes (except for Muller F" -- cyst and hub cells are somatic cell type and also show somehow lower expression than muller B and E. What would be the explanation for this?

Good question but we can only speculate. The dot of pseudoobscura group contains a translocation from the ancestral Y to the dot, which we suspect may cause unique/clade-specific regulatory features of the chromosome.

4) "Lower expression from the X in male tissues compared to female tissues has been interpreted as an adaptation of the X to female-biased transmission (i.e. feminization of the X)." -- If you remove male-biased or male-specific genes, does this pattern still hold?

To do this, we compare expression levels in male and female bulk RNA-seq, and identified genes as male-biased if the expression is >1.5-fold in males vs females. Removing these genes in the scRNA-seq data yielded higher X:A ratios (but was still below 1 at least for the younger X chromosomes). This may indicate that dosage compensation has not fully evolved on the younger X’s.

5) What are the genes associated with different burst rates? Do you think this pattern is random or selectively regulated? What would be the most exciting biology that tells us?

At the request of reviewer 1, we have removed the burst rate analyses.

6) The authors used the word "loss of dosage compensation" throughout the manuscript, and I wonder if there is a better word to describe this. By definition, during and after meiosis, cells become haploid and do not need to be compensated. Thus, rather than loss of dosage compensation, it is the shutdown of dosage compensation, unless the authors indicate that dosage compensation is already "lost" in spermatocytes. In addition, although I don't think the authors indicate it, the word "loss of dosage compensation" sounds as if there is an evolutionary loss of dosage compensation.

We changed this to “shut down” of dosage compensation.

7) Many of the references are wrongly labeled, such as "This mechanism of relative X downregulation is fully consistent with both the findings in Witt et al [30,34] and Mahadevaraju et al [35,36]." "Meiklejohn et al and Landeen et al that down regulation occurs across the testes and is not specific to meiosis [37],". "While this is consistent with previous reports in D. melanogaster testes scRNA-seq dataset [80,81], the germline is thought to lack dosage compensation in flies. This is primarily based on the cytological observation that many of the MSL components are absent in the germline, and the male germline appears to lack X-chromosome specific H4K16ac [81,82]." These are just a few examples of the many wrong citations. I recommend the authors recheck all the references in the whole manuscript.

We apologize about this. There was a glitch in the citation software that we overlooked. We have redone the references.

8) "the replicated scRNA-seq experiment yielded 10,990 cells that fall into 12 clusters (Figure 1D, Supplementary figure 1A). " - from the methods, it seems that the authors only kept ~5000 cells for further analysis. If so, please indicate it here.

Added.

9) It might be helpful to add gene names in figure 1D as a supplementary figure.

The list of genes that Seurat used to contrast between clusters is quite large and rather unwieldy. For identifying the cell types of the clusters, we recommend looking at Supplementary figure 1 which we have updated to include many more diagnostic markers.

**Response to Reviewer #3:**

The manuscript by Wei, Chatla, and Bachtrog describes experiments that measure gene expression in the testes of Drosophila miranda to understand the evolution of sex-chromosome regulation in the male germline.  They use single-cell RNA sequencing to identify cells in differing stages of spermatogenesis and compare expression of genes located on D. miranda's three X chromosome arms that have been X-linked for very different lengths of evolutionary time.  These three X-linked chromosome arms provide a very clear test to distinguish between two hypotheses that have been proposed to govern X-linked gene expression in the Drosophila testes: MSCI vs. a loss of dosage compensation.  Under the model that MSCI is an adaptive gene regulatory process that is gradually co-opted by chromosomes after they become X-linked, the old X-linked arms should show the most MSCI, while the younger arms might still be in the process of acquiring meiotic silencing.  In contrast, the alternative model that posits the relatively lower expression from the X chromosome in male meiosis is a consequence of the absence of dosage compensation in these cells.  Under this model, the reduced expression of X-linked genes in the male germline is a simple consequence of their ploidy, and all X-linked chromosome arms, regardless of age, should show a similar ~1.5-2-fold reduction that has been observed in other contexts where dosage compensation is missing.  The experiments presented in this manuscript unambiguously support the second model, as they reveal that the youngest X-linked arms have the lowest expression across meiosis, and that in all cases reduced X-chromosome expression is at most ~50% of autosomal expression. 

I found the manuscript clear, easy to read, and I think its conclusions are supported by the results.  I have only mostly minor comments for the authors.

In figure 2A, the bars in the "Loss of DC" panel show a very slight decrease in expression with decreasing X-chromosome age, and the manuscript on page 5 states "if reduced meiotic expression simply reflects loss of dosage compensation instead of targeted activation, opposite expression patterns are expected".  I infer that the "opposite" here reflects the slight decrease with decreasing age, in contrast to the MSCI prediction of decreasing expression with increasing age.  However, I didn't see any prediction or theoretical expectation in the manuscript for why we expect to see this "opposite" pattern of decreasing expression with decreasing age.  This should be cleared up - if the prediction for loss-of-dosage-compensation is equal expression across X-linked arms, then this should be explicitly stated and the "Loss of DC" panel in Figure 2 should be modified so the expression of across age is equal.  If by "opposite" the authors do mean that they expect higher expression associated with older X-linked arms, this expectation should be explained and I suggest making the differences between the bars in the "Loss of DC" panel easier to see.

We meant the former (equalization of the three Xs), and have changed “opposite” to “distinctly different”. We have also corrected the last panel of 2A to reflect equalization.

If I am interpreting Figure 2B correctly, each point that goes into the bar graph is a single cell?  This means that the X-autosome ratio of expression varies from at least (the range of the data is cutoff in the graph, which is not ideal) 4-fold over-expression of the X to 8-fold over-expression of autosomal genes across cells at the same stage of spermatogenesis.  Is this level of heterogeneity common in single-cell RNAseq experiments?  Is there anything interesting to say about this variation?  For example, if one were to compare two cells of the same stage with wildly different X:A expression ratios, would the differences in those ratios be driven by the same genes expressed in those cells, or because they are expressing different genes from each other?

Here the single cell counts were summed for each cell type, such that for each gene, we have an aggregate value for each cell type. The boxplots represent the distribution of gene expression normalized by the autosomal median for each chromosome and cell type. So the variance is not cell to cell variance, but expression differences of different genes. This is why the range of expression is substantial, as expected with the range of expression for different genes. We have made this more clear in the figure legends. Because the range of DC vs no DC is rather small (between –1 and 0 of log2(X:A)), we have to zoom in to really show the subtle but significant and diagnostic differences between the chromosomes.

Line numbers make reviewing manuscripts much easier.  I ask the authors to consider the courtesy of adding them for reviewers in the future.

Added.

Page 2: "resulting in the multinucleated cysts called the spermatocytes" - everything I have read refers to each individual nucleus as a spermatocyte (16 spermatocytes in a cyst), rather than all 16 comprising a single spermatocyte.  Is this common terminology?

This is an error on our part. Thank you for catching it.

Page 6, top: "equalizing at meiotic exist" should be "exit"

Corrected

Page 6: looking at Supplementary Figure 3, I was not convinced that the autosomes have elevated burst sizes.  I think statistical justification for this claim should be presented.

We have removed the transcriptional burst/kinetics analyses, as reviewer 1 pointed out that underlying assumption to the model is not met.

Page 6, second section: The citations for ChIP-seq data (67 and 68) are incorrect I think.

Corrected.

Page 10: I think the manuscript should state that ribosome profiling was conducted using 3rd instar larvae, not testes.

Noted.

Page 10, second section: I think "over-represented with genes highly expressed in the testes" would be clearer as "over-represented among genes highly expressed in the testes"

Corrected.

Page 11, second paragraph: last sentence should include "genes" or "chromosome" after "neo-Y"

Added “genes”

Page 14: there are citations missing where it states "Meiklejohn et al" and "Landeen et al"

Corrected.

Page 14: I have always found the pole cell transplant experiments in Bachiller and Sanchez 1986 (<https://pubmed.ncbi.nlm.nih.gov/3098595/>) one of the most compelling experiments demonstrating that msl-1 and msl-2, but not mle, are dispensable for male fertility.  The authors might consider including that citation.

Added.

Page 15, top: open chromatin on the X is more strongly associated with what?

Corrected to “Further, a recent comparison of the nuclear topology of germline stages reported that in the spermatogonia and spermatocytes, active chromatin regions on the X have more intra-chromosome contacts than the autosomes potentially suggesting active regions on the X are gathering at transcription-rich nuclear territories allowing for higher expression”.

Page 16: The absence of citations in the last paragraph of the discussion seemed odd, as the theory and data presented in that paragraph all could use citations.

Citations have now been added.

In figure 4, I have a very hard time distinguishing the MSL-proximal and MSL-distal points

We have increased the contrast of the two types of points.


**Response to Academic Editor**:

Three expert reviewers in the field have evaluated your manuscript. As you can see, there was considerable disagreement among the reviewers on the rigor of your study and by extension the strength of the conclusions.

A major experimental concern expressed by reviewer 1 is that you did not cytologically validate that testes markers from D. melanogaster are expressed in similar cell types or stages in D. miranda. If you have already performed these validations and have data available, then I encourage you to include them in your resubmission. However, if you have not already performed a cytological examination of marker genes in D. miranda testes then I do not think these experiments are required. I do however suggest that you acknowledge this assumption in your study and address any impact it may have on your conclusions.

We do not have any cytological validations available, however we are also unsure whether cytology would be as definitive as reviewer 1 make it seem (see our response above). We have added in text the potential differences between spermatogenesis in D. melanogaster and D. miranda. We also added additional marker genes to show that we are properly identifying germline stages.

Reviewer number 1 also expresses concern that certain assumptions critical for the transcriptional kinetic analysis implemented in Figure 2C-E are not met during spermatogenesis. It is important that you address whether these assumptions are met and if not, how this might impact your conclusions. If upon further reflection you decide it is not appropriate to implement these analyses then I think you could remove them without compromising the major conclusions of your paper.

The burst kinetics analyses have been removed.

In addition to these concerns the three reviewers have many concerns and suggestions that should be addressed in your point-by-point response to review. In addition to the reviewers, I would like to add a few suggestions of my own:

1) I think you are making a valuable new addition to our understanding of X chromosome regulation Drosophila male meiosis. However, as reviewer 1 notes, this is a very contentious field and, as evidenced by the diverse responses of the three reviewers, many readers may be inclined towards skepticism of your work. In this case I think it is extra important that you are thorough in your discussion of findings and limitations of previous work and what new insights are brought by D. miranda biology and your study. For example, in the introduction you rightfully point out that sex chromosome evolution can be a major confounding factor in studying sex-chromosome specific regulatory regimes, since the feminization of X chromosomes reduces X-gene expression similarly to loss of dosage compensation or MSCI. This is one major benefit of studying D. miranda, since in addition to having less time to acquire dosage compensation or MSCI, the neo-X chromosome has had less opportunity to become feminized. I think this point could be stronger in the introduction.

We have added these suggestions to the introduction and additionally emphasized the unique opportunity of looking at a young X chromosome and the possibility of comparing to the autosomal orthologs (in D. psuedoobscura) to infer the ancestral regulatory states. For this revision, we have specifically strengthened our inferences and conclusions by comparing the expression of lowly expressed (and MSL-distal) genes to their autosomal orthologs, and Y-gametologs, and concluded that the low expression reflects ancestrally low expression, instead of acquired suppression after sex-linkage. These new analyses can be found in the results section under the headings “Distinct germline transcriptional trajectories depending on MSL proximity” and “Low meiotic expression of a subset of neo-X genes reflects ancestral expression “.

2) Related to point 1, a lot of skepticism about your work centers around the observations of Mahadevaraju et al 2021, which shows that there are chromosome territories in D. melanogaster larval male meiosis that may repress transcription from the X and 4 chromosomes by regulating pol II. With the caveat that the study in is in D. melanogaster larvae, which could differ substantively from D. miranda adults, Reviewer 1 rightfully points out that if MSCI is governed by nuclear chromosome territories, the physical linkage between the neo and ancestral X could be sufficient to establish MSCI equally across ancestral and neo X chromosomes, which is a pattern similar to that which you observe. I think it is important that you allow for this scenario when setting up your predictions in Figure 2A. There is other data you can draw upon to support your argument that your data are most consistent with loss of dosage compensation.

We believe that we have sufficiently addressed/rebutted all the points that reviewer 1 raised, and revised the text accordingly. Importantly, we have discussed (in the rebuttal as well as the manuscript text) how the cytological data from Mahadevaraju et al 2021 is consistent with the loss of DC. The same conclusion was reach by Anderson et al (eLife 2023), who used chromatin profiling to study active Pol-II in *D. melanogaster* spermatogenesis, and they stated that observed levels of active Pol-II are consistent with loss of DC. This study is now discussed in our manuscript (p. 17).

Regarding the possibility of incorporation of the neo-X into the existing repressive territory, we find this model to be untenable when considering how neo-sex chromosomes evolve. First, note that the neo-X (Muller C) is not physically linked with the older X chromosome arms which tempers the possibility of this model in *D. miranda*, as Muller C would need to acquire an incorporation mechanism. Second, as we discussed above, if physical linkage is sufficient to induce immediate/rapid incorporation of the neo-X into a repressive territory, neo-X chromosomes (through fusions) would never form because they would be immediately silenced in the germline/meiosis, leaving expression from only the Y-linked (or proto/neo-Y-linked) gametolog. For example, in the species *D. albomicans*, an autosome over 55Mb was fused with the X, creating a neo-X. Since aneuploidy of any autosomes (other than the dot) in Drosophila leads to lethality, it seems extremely unlikely to us that meiosis or any cellular process could properly proceed when over 1/3 of the genome is dosage imbalanced. Indeed, we examined the testes RNA-seq expression of the very young neo-sex chromosomes in *D. albomicans* and find a slight neo-X bias indicative of early gene-by-gene degeneration instead of X-inactivation through physical linkage and incorporation into repressive territory. Therefore, MSCI acquisition seems only feasible if it is a gradual process (like other sex chromosome features) with commensurate upregulation from Y gametologs or gene movement off the X.

Minor comments:

1) On page 5 your claim that y chromosome genes are disproportionately upregulated upon meiotic exit, you need to provide some statistics to support this claim or change the language.

This was initially only in the supplementary figures. It is now added to the text.

2) Page 9 “in lieu of” means “in place of” which I don’t think is appropriate in this sentence. Maybe “in the face of” would be a more appropriate phrase?

Corrected to “in light of”.

3) Page 10 you state that pseudogenized Y transcripts are likely to be degraded rather than translated. You have the data to evaluate this expectation in your system, is it true?

This is the case. We have noted this and added this as supplementary figure 10A, showing that neo-Y genes with truncated CDSs have significantly less ribosome profiling reads (reduced by about 4-fold).

4) Page 11: you state the the Y is “losing testes genes to the Y” chromosome. I understand what you are saying but I find the language problematic because it implies the genes are physically relocating, which they are not.

Changed to “These results reveal that the X is becoming demasculinized through preferential loss of testes genes thus becoming neo-Y-specific".

5) The co-amplified genes on the X and Y are really interesting, however their roles in meiotic drive are entirely speculative as you acknowledge. I wonder whether some of the speculative discussion would not be better moved from the results to the discussion.

We agree that the middle paragraph could be moved to the discussion, but personally prefer it where it is. This can be changed, however.

Anderson J, Henikoff S, Ahmad K. Chromosome-specific maturation of the epigenome in the Drosophila male germline. eLife. 2023. doi:10.7554/elife.89373

Conrad, Thomas, Florence M. G. Cavalli, Juan M. Vaquerizas, Nicholas M. Luscombe, and Asifa Akhtar. 2012. “Drosophila Dosage Compensation Involves Enhanced Pol II Recruitment to Male X-Linked Promoters.” *Science* 337 (6095): 742–46.

Ferrari, F., Y. L. Jung, P. V. Kharchenko, A. Plachetka, A. A. Alekseyenko, M. I. Kuroda, and P. J. Park. 2013. “Comment on ‘Drosophila Dosage Compensation Involves Enhanced Pol II Recruitment to Male X-Linked Promoters.’” *Science*.

Ferrari, Francesco, Annette Plachetka, Artyom A. Alekseyenko, Youngsook L. Jung, Fatih Ozsolak, Peter V. Kharchenko, Peter J. Park, and Mitzi I. Kuroda. 2013. “‘Jump Start and Gain’ Model for Dosage Compensation in Drosophila Based on Direct Sequencing of Nascent Transcripts.” *Cell Reports* 5 (3): 629–36.

Straub, Tobias, and Peter B. Becker. 2013. “Comment on ‘Drosophila Dosage Compensation Involves Enhanced Pol II Recruitment to Male X-Linked Promoters.’” *Science*.

Vaquerizas, Juan M., Florence M. G. Cavalli, Thomas Conrad, Asifa Akhtar, and Nicholas M. Luscombe. 2013. “Response to Comments on ‘Drosophila Dosage Compensation Involves Enhanced Pol II Recruitment to Male X-Linked Promoters.’” *Science*.
